# Application of Mass Spectrometry in Pancreatic Cancer Translational Research

**DOI:** 10.3389/fonc.2021.667427

**Published:** 2021-10-11

**Authors:** Peng Ge, Yalan Luo, Haiyang Chen, Jiayue Liu, Haoya Guo, Caiming Xu, Jialin Qu, Guixin Zhang, Hailong Chen

**Affiliations:** ^1^ Department of General Surgery, The First Affiliated Hospital of Dalian Medical University, Dalian, China; ^2^ Institute (College) of Integrative Medicine, Dalian Medical University, Dalian, China; ^3^ Laboratory of Integrative Medicine, The First Affiliated Hospital of Dalian Medical University, Dalian, China

**Keywords:** pancreatic cancer, mass spectrometry, translational science, diagnosis, therapy

## Abstract

Pancreatic cancer (PC) is one of the most common malignant tumors in the digestive tract worldwide, with increased morbidity and mortality. In recent years, with the development of surgery, chemotherapy, radiotherapy, targeted therapy, and immunotherapy, and the change of the medical thinking model, remarkable progress has been made in researching comprehensive diagnosis and treatment of PC. However, the present situation of diagnostic and treatment of PC is still unsatisfactory. There is an urgent need for academia to fully integrate the basic research and clinical data from PC to form a research model conducive to clinical translation and promote the proper treatment of PC. This paper summarized the translation progress of mass spectrometry (MS) in the pathogenesis, diagnosis, prognosis, and PC treatment to promote the basic research results of PC into clinical diagnosis and treatment.

**Graphical Abstract:**
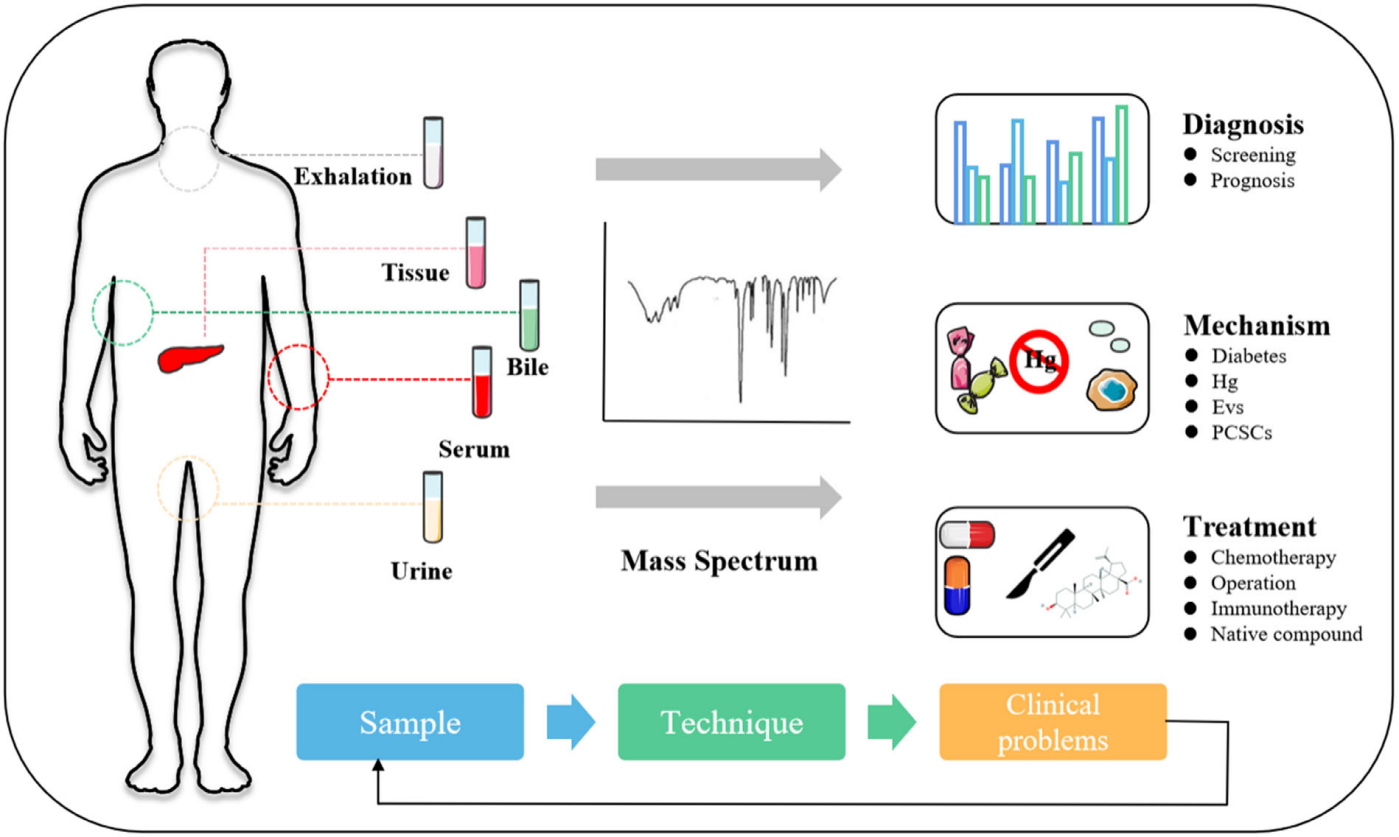


## 1 Introduction

Pancreatic cancer (PC) is an aggressive malignant tumor with 95% pancreatic ductal adenocarcinoma (PDAC). Due to the lack of specific early symptoms, most patients had already reached the advanced stage when they visited the physician and lost the opportunity to undergo surgery (1). Moreover, even though PC patients were undergoing surgical resection, the prognosis may worsen, with a recurrence rate of over 80% in 2 years (2). After entering the 21st century, with the rapid development of medicine, basic and clinical studies such as liquid biopsy, marker screening, targeted therapy, and immunotherapy for PC emerge one after another (3, 4). However, the incidence and mortality of PC increased gradually, without reduction, and the 5-year survival rate was only 7% (5). This contradiction indicates that some gaps may exist in basic and clinical research to address clinical problems. Fortunately, translational science can be a workable strategy that offers new opportunities for the dilemmas of basic and clinical research on PC.

Translational science, a theory in development that links basic research to clinical treatment, highlights the bi-directional link between the laboratory and the hospital bed. Since this concept was formally put forward by the National Institutes of Health (NIH) in 2003, this emerging medical field has become a critical link between essential medicine and clinical practice. We quote here the explanation of translational science by Austin CP, Director of the National Center for Advancing Translational Sciences (NCATS) (6). First, we need to make clear the difference between translational science and translational research that we are familiar with; translational research is the driving force of clinical research, focusing on specific diseases or problems; translational science is a universal translation process, whose core lies in improving the efficiency of translation. Second, translational science is underpinned by translational scientific research for its advancement. This research is based on the “clinical–laboratory–hospital bed” paradigm with a focus on process integrity. Third, basic and translational scientific research complement one another and are inseparable from the resolution of the “general problems” of “general diseases” (6). Acting on the principle of translational science, researchers can see the dawn of the resolution of the PC’s problem. We should start with the troublesome PC problems found in the community and hospitals and find the proper technology in the laboratory to address these clinical problems. Recent studies have suggested that mass spectrometry (MS) can play a vital role in PC translation. In this review, we will present the research progress of MS in PC diagnosis and treatment.

## 2 The Characteristics of Various MS Methods

MS analysis is a powerful tool to identify pure substances, including measuring relative molecular weight, determining chemical formula, and identifying structure. The world’s first mass spectrogram was recorded by physicist J.J. Thomson. Later, F.W. Aston and A.J. Dempster developed universal approaches of MS. Over time, MS tools and technologies have been constantly improved and developed. Since the 1980s, MS has been gradually used in life science research and pharmaceutical industry. Today, the value of MS in therapeutic drug monitoring, genetic disease screening, vitamin series detection, immunology, microbiology, and other medical fields is widely acknowledged by researchers (7).

### 2.1 Chromatography-MS

Mass spectrometers are often used in combination with chromatographic techniques for improved separation of complex samples before analysis. Among them, liquid chromatography (LC)-MS and gas chromatography (GC)-MS are the most widely adopted chromatography-MS techniques (8). **Table 1** presents the main characteristics of LC-MS and GC-MS.

**Table 1:** The characteristics of GC-MS and LC-MS.

Methods	GC-MS	LC-MS
Main characteristic	Mature and stable	High sensitivity/10^−15^ (g)
	Small sample used	Small sample used
Applied range	Limited	Wide
	Volatile	Most metabolites
	heat-stable substance	
Sample treatment	Tedious	No special treatment is required
	Derivatization is required	
Costs	General	More expensive than GC-MS
Database	Available	Limited
	NIST, Fiehn, GMD	METLIN, MassBank, mzCloud

#### 2.1.1 GC-MS

GC-MS is a method that combines the features of GC and MS and analyzes a range of components. GC-MS can determine the molecular structure of compounds, accurately determine the molecular weight of unknown ingredients, and determine low concentrations of substances. It offers clear advantages: low detection limit, high sensitivity, greater qualitative and quantitative accuracy, etc. (9). As a non-invasive screening, volatile organic compounds (VOCs) have a significant clinical application perspective for the early diagnosis of cancer. Currently, the GC-MS is the benchmark for comprehensive VOC analysis. Many studies have reported the benefits of GC-MS in the search for tumor patient VOC biomarkers (10, 11). In addition to VOCs, GC-MS is also widely used to detect metabolites in cancer patients’ plasma and urine samples. With the assistance of GC-MS analysis and machine learning, Wang et al. obtained three potential biomarkers (L-serine, myo-inositol, and decanoic acid) to distinguish prostate cancer (PCa) from benign prostatic hyperplasia (12). Another study used GC-MS to identify five lipids as potential serum biomarkers in breast cancer diagnosis (13). Recently, GC-MS has been used for the non-targeted lipidomics analysis of formalin-fixed, paraffin-embedded (FFPE) tissue samples. Specifically, the researchers found that formalin has no significant effect on the presence of specific metabolites in cancer tissue by GC-MS (14). Later, Buszewska-Forajta et al. identified three fatty acids (FAs) in FFPE as potential markers for the diagnosis of PCa (15). In short, GC-MS provides a new idea and approach for the study of cancer biomarkers.

#### 2.1.2 LC-MS

LC-MS is the most widely adopted technical platform in metabolomics research with the advantages of high sensitivity, robustness, and good metabolite coverage. Based on LC-MS, the combination of ultra-performance liquid chromatography (UPLC) and high-resolution MS can strengthen the resolution, sensitivity, and flux of assembly analysis data. Reversed-phase liquid chromatography (RPLC) is mainly used to separate non-polar or medium-polar metabolites and has a strong separation and detection capability (16). LC-MS is more sensitive than GC-MS because they can analyze strongly polar, non-volatile, and thermally unstable compounds, and GC-MS is not up to it. For metabolomics, LC-MS is suitable to detect and analyze most of the metabolites (up to 10,000). However, LC-MS lacks a full standard spectral library similar to GC-MS. The identification of compounds is a time-consuming task that requires additional time and effort from researchers (17, 18).

Among the strategies for analyzing the proteome’s expression, structure, and function, LC-MS has played and will continue to take a vital role (19). On the one hand, high-throughput proteomics based on LC-MS has been widely used to discover tumor biomarkers. LC-MS can provide potential biomarkers for early diagnosis, prognosis prediction, and chemotherapy resistance of cancer; on the other hand, LC-MS analysis can offer potential therapeutic targets for treating tumors and then perform targeted therapy precisely.

LC-MS, a powerful technology, propels proteomics research towards a new era. By contrast, traditional protein identification methods, such as Western blotting, are time-consuming and inefficient, and unsuitable for high-throughput proteome studies; enzyme-linked immunosorbent assay has been widely used in clinical and environmental laboratories, but it is not sensitive enough to detect proteins with low concentration or weak signal. MS can be used for qualitative identification and quantitative analysis of proteome in a comprehensive and systematic manner, and its sensitivity and detection limit are well above traditional methods (20). Of course, the disadvantages of MS are also apparent, including complex operation procedures, poor reproducibility, and high cost.

### 2.2 MALDI

Recently, novel methods for ionization have been applied to MS. Matrix-assisted laser desorption ionization (MALDI)-MS is an efficient soft ionization MS technology. Its combined application with a time-of-flight (TOF) mass analyzer provides a marvelous tool for analyzing mixtures and biological macromolecules, possessing the advantages of high accuracy, wide detection range, and good compatibility. MALDI-TOF-MS has been developed in genotyping analysis, biomarker identification, pathogen identification, drug metabolism, and MS imaging (MSI) and is increasingly favored by the clinical detection field of view (21). MALDI-TOF MS is suitable for determining proteins, peptides, polysaccharides, nucleic acids, and other macromolecular substances with a relative molecular weight of 1,000–3,000 Da and can quickly and effectively detect serum, plasma, urine, and other body fluids. Specifically, MALDI-TOF MS is capable of directly determining unseparated macromolecular mixtures. It should be noted that MALDI-TOF MS cannot effectively identify small molecular peptides with a relative molecular weight of less than 500 (22). Unlike MALDI-TOF-MS in the detection of biological macromolecules, laser ablation-plasma MS (LA-ICP MS) is more widely used to determine the elemental composition of natural and synthetic materials. It has significant advantages in the analysis of trace and ultra-trace elements, especially in rare earth elements, platinum family elements, and isotope analysis. Probe electrospray ionization (PESI) is a new environmental ionization technique that uses an excellent, low-invasive needle to collect a few milligrams of tissue for direct MS analysis (no pretreatment is required). Compared to traditional methods, PESI-MS is simple to operate and requires no maintenance. It may be used as a potential screening method for individuals at high risk of cancer.

### 2.3 MSI

MSI, a fast-growing molecular imaging technology, visually analyzes the structure, spatial and temporal distribution of molecules in cells or tissues using MS and the associated imaging software (23). In decades, MSI technologies based on MALDI, atmospheric pressure-MALDI, desorption electrospray ionization (DESI), and secondary ion MS (SIMS) ion sources have developed rapidly and had potential value in tumor diagnosis, marker screening, and treatment (24). The visual MALDI-MSI developed from MALDI-TOF MS can analyze biomolecules *in situ* and provide spatial distribution information (without the use of antibodies). MALDI-MSI is currently the most widely used MS imaging technology to analyze small molecular metabolites, lipids, peptides/proteins, and small drug molecules with the help of various substrates. The molecular spatial distribution information provided by MALDI-MSI may directly show the changes in substances under pathological conditions. Traditional methods such as immunohistochemistry are widely used to show the spatial distribution of specific enzymes in tissues based on antibody–antigen binding. Nevertheless, this method relies on targeted antibodies, takes time, and cannot be untargeted for unknown characteristics. MSI can directly map metabolites, enzymes, and lipids related to tumor genesis and development without labeling or molecular printing, thus obtaining spatial information of biomarkers, drug metabolism, and tissue types (25).

## 3 Hinting on the Pathogenesis of PC by MS

PC is a complex molecular biological process involving multi-genes, multi-signal pathways, and multi-steps (26). Based on the rapid development of gene sequencing technology, the researchers discovered that PC is accompanied by massive genetic mutations (including K-ras, TP53, and CDKN2A) (27), which offer the potential for the occurrence of cancer. Moreover, the tumor microenvironment provides a hotbed for cancer development and promotes tumor immune escape (28). However, the role of several internal and external factors in the pathogenesis of PC has yet to be resolved.

### 3.1 Is Preoperative Biliary Drainage Necessary?

Malignant obstructive jaundice is one of the most common clinical complications among PC patients. Cholestasis caused by pancreatic space-occupying lesions could damage liver and kidney function. However, the need for biliary drainage before the operation for PC-obstructive jaundice has been controversial due to severe complications (29). Gál et al. adopted LC-MS to determine serum bile acid (BA) concentration in PC patients and explored various analytical methods to confirm the role of BAs on tumor progression. They found that BAs increased the tumorigenicity of PDAC cells by overexpressing Mucin-4 (MUC4) (30). MUC4, a member of the mucin family, is characterized by membrane-bound and secretory proteins. It is difficult to find MUC4 in normal pancreatic tissue from the gene expression level, while MUC4 is significantly overexpressed in PC. In terms of mechanism, MUC4 promotes the occurrence and metastasis of PC by regulating the epithelial–mesenchymal transformation and interfering with the function of the immune cell (31). Gál et al. demonstrated a significant increase in serum BA level in PDAC patients. Also, the increased level of serum BAs increases the expression of MUC4 in the PC, which may accelerate the progression of the tumor (30). Based on the above results, early relief of obstructive jaundice may benefit PC patients with indications, although more prospective randomized controlled trials are required to verify its efficacy.

### 3.2 Risk Factor

Massive studies have described the bi-directional relationship between diabetes and PC. On the one hand, diabetes dramatically increases the risk of PC and affects the survival status of PC patients (32); on the other hand, diabetes is also a paraneoplastic phenomenon caused by PC (33). Unfortunately, the specific mechanism of the interaction between diabetes and PC is not fully understood, which is not conducive to the development of strategies for early detection of PC in people with diabetes.

Velazquez et al. expect to use transcriptome and metabolome changes to reveal the mechanism of diabetes that promotes PC growth and invasion (34). They found that type 2 diabetes mellitus accelerates cancer growth by driving metabolic reprogramming (mainly kynurenine, tryptophan, taurine, and choline pathways), whereas metformin therapy reverses metabolic reprogramming to inhibit cancer growth. In general, the metabolite curve determined by MS provides strong evidence to reveal the causal relationship between diabetes and PC.

With the worsening of environmental pollution, individuals with excessive heavy metals in their bodies are often found. Even low concentrations of heavy metals can damage DNA, RNA, and protein, leading to cell apoptosis, cell cycle changes, and cell carcinogenesis (35). As such, we cannot ignore the role of heavy metals in the occurrence and development of cancer. Mercury, a genotoxic metal, contributes to the pathogenesis of many cancers, including colorectal and brain tumors (36, 37). However, the role of mercury in the pathogenesis of human PC has not been explained. Pamphlett et al. adopted autometallography (a sensitive amplification technique for detecting mercury sulfide/selenium molecules) and LA-ICP-MS to determine the distribution of mercury in pancreatic cells isolated from patients with PC and normal volunteers (38). They found that mercury is mainly present in islet cells of patients with PC, which may be linked to the distribution of abundant porous capillaries around the islets. Like other toxic heavy metals, mercury can promote the development of PC by affecting auto-immunity and oxidative damage. Interestingly, the content of mercury in islets does not match age. In summary, the findings based on auto-metallography and LA-ICP-MS may support the hypothesis that mercury leads to the pathogenesis of PC.

### 3.3 Extracellular Vesicle-Mediated Cancer Phenotypes

Extracellular vesicles (EVs) are nanometric particles secreted by different cells and essential for intercellular communication. Increased studies have shown the role of EVs in promoting cancer proliferation, metastasis, and resistance. One study reported that microvesicles derived from human PC cells encourage tumor cell proliferation, invasion, and metastasis by activating the PI3K/Akt/mTOR pathway (39). Several of the reviewed studies suggest that EV proteins or non-coding RNA may serve as biomarkers for the diagnosis and prognosis of PC (40). In short, EVs are the most promising biomarker and therapeutic application of PC. The profiles of hydrophilic metabolites in EVs have also been recently mapped (41). In one study, the hydrophilic metabolome of small EVs (sEVs) and PANC-1 cells (human pancreatic cancer cell line) were analyzed by capillary IC-MS and LC-MS. Indeed, 11 hydrophilic metabolites were identified only in sEVs. Inosine, guanosine, and xanthine, which are involved in purine metabolism, and cytidine and uridine, which are involved in pyridine metabolism, may create an appropriate environment for proliferation and metastasis of PANC-1 cells *via* immunosuppression. Furthermore, hypoxic stress may affect the content of sEVs metabolites by modifying the PC cell amino acid metabolism. The study also examined differences in lipid content sEVs and cells by supercritical fluid chromatography coupled with triple quadrupole mass spectrometry (SFC-QqQMS). The researchers found that triglyceride decreased while monoacylglycerol and diacylglycerol increased significantly in sEVs. Surprisingly, hypoxic stress had only a weak effect on the lipid content in EVs. In conclusion, this study provides a preliminary map of the differences in hydrophilic metabolites and lipids in sEVs, revealing a potential mechanism for tumor growth and metastasis implication.

### 3.4 Pancreatic Cancer Stem Cells

Pancreatic cancer stem cells (PCSCs) are a significant focus of PC research, as this subset of cancer cells plays a crucial role in tumor metastasis and resistance to anti-cancer drugs. Recent studies have suggested that targeting signaling pathways in PaCSCs (including Notch, Wnt, Hippo, and AKT/mTOR) can significantly improve the effectiveness of chemotherapy. Targeting miRNAs to regulate PCSCs has also been investigated to regulate clinical chemoresistance. However, the molecular phenotype of PCSCs has not been fully identified from a systems biology point of view.

One study identified the modulated pathways of PCSCs *via* iTRAQ-based proteomic analysis, revealing that PCSCs changed proteomic and metabolite profiling compared to Panc1 parental cells. FAs and mevalonate play pivotal roles in the survival of PCSCs (42). Another study used LC-MS/MS to examine the protein modulation and lipid modulations in four PCSCs (43). Results showed that most of the upregulated PCSC proteins belonged to mitochondrial proteins associated with metabolic pathways and lipid metabolism. Analysis of KEGG enrichment revealed that the differential proteins were related to FA elongation and unsaturated FA biosynthesis. The lipid profile showed that long-chain FAs and super long-chain FAs in PCSCs were increased. Further analysis predicted that phosphoinositol signal transduction, FA extension, and cardiolipid acyl chain composition alterations are the specific signal transduction pathways of PCSCs.

Therefore, MS has proven useful in elucidating PC’s pathogenesis and the regulatory role of EVs or PCSCs in its development, providing future guidance for the diagnosis and treatment of PC.

## 4 Application of MS in the Diagnosis and Prognosis of PC

**Figure 1:**
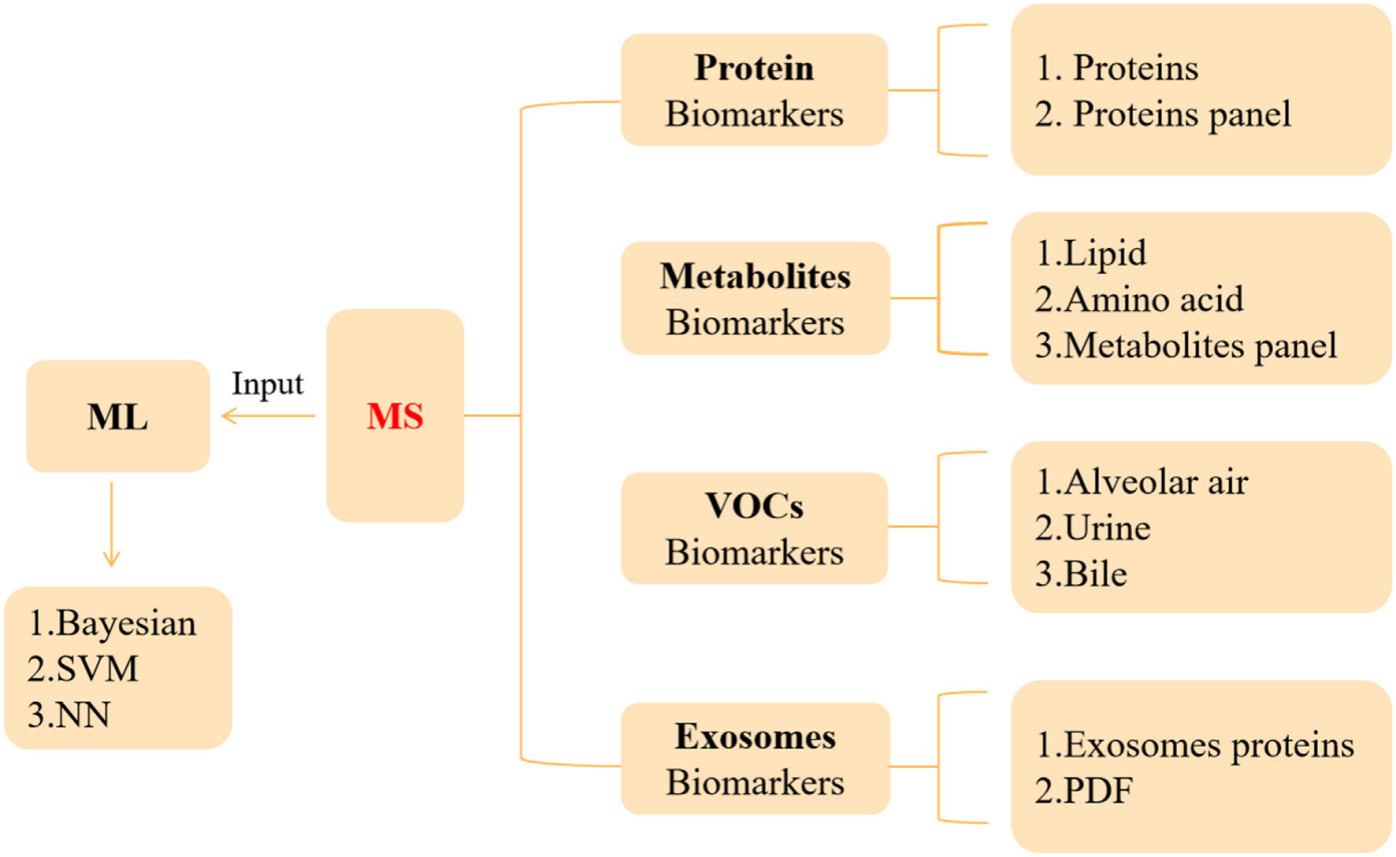
Application of MS in the diagnosis and prognosis of PC.

The development of PC is hidden, and the clinical diagnosis mainly depends on clinical manifestations, color ultrasound, CT, magnetic resonance, and other imaging examinations (44). It is with regret that most patients were diagnosed in the middle and late stages. Therefore, the determination of an effective and accurate early screening strategy is helpful to improve the detection rate and survival rate of PC. This section will describe the auxiliary role of MS in serum marker carbohydrate antigen 19-9 (CA19-9), VOCs, metabolites, exosomes, and artificial intelligence (AI) in the diagnosis and prognosis of PC (**Figure 1** and **Table 2**).

**Table 2:** Novel biomarkers of pancreatic cancer using MS.

Sample	Type	MS Method	Biomarker	Significance	Reference
Serum	Bile acids	LC-MS	GCA, TCA, GCDCA,	Bile acids accelerate tumor processes.	Gál et al. (30)
Serum	Glycan	MALDI-MS	TCDCA, IgG Gal ratio	A novel biomarker that can be used in PC patients with negative CA19-9 levels.	Zhong et al. (45)
Tumor tissue	Secretome	NanoLC-Tandem-MS	THBS2	Combined expression of THBS2/CA19-9 could accurately distinguish patients with PDAC from HC.	Le Large et al. (46)
Plasma	Protein	LC-MS/MS	APOA4, CLEC3B, GSN, SERPINF1	Multiple biomarker panels can facilitate early detection of PDAC in diabetic patients.	Peng et al. (47)
Urine	Protein	GeLC/MS/MS	LYVE-1, REG1A, TFF1	Early detection of PC	Radon et al. (48)
Plasma	Glycopeptides	LC-MS/MS	fucosylated SERPINA1	A novel prognosticator for PC	Wu et al. (49)
Exhaled breath	VOCs	IMR-MS	—	Specific VOCs composition can be used for clinical diagnosis of PC.	Princivalle et al. (50)
Urine	VOCs	GC-IMS/GC-TOF-MS	2,6-Dimethyl-octane, nonanal, 4-ethyl-1,2-dimethyl-benzene	The VOC composition of urinary headspace can be used to distinguish patients with PC from CP.	Arasaradnam et al. (51)
Bile	VOCs	—	ammonia, acetonitrile, trimethylamine	Measurement of VOCs in bile helped to accurately distinguish PC from CP.	Navaneethan et al. (52)
Plasma	Metabolites	UPLC-QTOFMS	Glycerophospholipids	Seven glycerophospholipids were associated with the risk of PC	Shu et al. (53)
Serum	Metabolites	RPLC-HRMS	Linoleic acid, glycerolipid, glycerophospholipid, primary bile acid	Metabolites can be used to distinguish unresectable PDAC patients from HC.	Martín-Blázquez et al. (54)
Plasma	Metabolites	GC-MS	Lysine	Metabolites are useful for detecting high-risk IPMN	Nakano et al. (55)
Tumor tissue	Amino acid	LC-MS/MS	Tissue amino acid index	The TAAI could serve as an independent prognosticator for patients with PDAC	Hiraoka et al. (56)
Pancreatic duct fluid	Exosomes	LC-MA	CEACAM 1/5	Exosomal proteins may be utilized to diagnose patients with PDAC	Zheng et al. (57)
Serum	Metabolites	UPLC-Q/TOF-MS	3-Hydroxyadipic acid, d-galactose, LysoPC (P-16:0), tetradecenoyl-l-carnitine	Metabolites could be effective predictive indicators of the efficacy of chemotherapy in PC patients.	Wu et al. (58)
Tumor tissue	—	LC-MS/MS	Vestigial-like 1	VGLL1 was inversely correlated with the survival of patients with PDAC	Bradley et al. (59)

### 4.1 Protein Biomarkers

CA 19-9, the most widely used gold-standard marker of PC, remains the only diagnostic marker of PDAC approved by the U.S. Food and Drug Administration (FDA) (44, 60). In a meta-analysis of 2,283 patients, CA 19-9 had a median sensitivity and median specificity of 79% and 82% for the diagnosis of PDAC, respectively (61). Ballehaninna et al. reported that the sensitivity and specificity of serum CA 19-9 levels in the diagnosis of PC in symptomatic patients are 79%–81% and 82%–90%, respectively. Moreover, serum CA 19-9 levels may also provide clinical significance on prognosis, overall survival, response to chemotherapy, and post-operative recurrence prediction (62). Fahrmann et al. recently reported that CA 19-9 could be an anchor marker for detecting early PC (63). In addition to using single CA 19-9 tests, researchers are always seeking additional biomarkers with CA 19-9 added value to better stratify the patients and guide therapeutic choices.

#### 4.1.1 IgG

IgG is a primary immune globulin in humans. Studies have shown that aberrant IgG glycosylation is strongly associated with cancers. Previously, Ren et al. adopted MS for the assessment of quantitative changes of IgG glycosylation in 114 non-cancer controls and 580 cancer patients. They found that the IgG–Gal ratio can recognize early PC (64), and then, they adopted MALDI-MS to map the serum IgG N-glycan profiles of 385 CA 19-9-negative serum samples. The results suggested that the IgG–Gal ratio can assist CA 19-9 diagnose early PC (the AUC of the IgG–Gal ratio combined with CA 19-9 reached 0.913) and effectively reduce clinical misdiagnosis (45). These findings suggest that the IgG–Gal ratio can be useful for early detection of CA19-9 negative PC patients. Further detailed clinical studies on samples remain necessary. Besides IgG glycosylation, serum fibrinogen may be a novel biomarker for the diagnosis and prognosis of PDAC. The overall sensitivity and specificity of serum fibrinogen in identifying PDAC patients were 67.4% and 83.6%, respectively, and the cutoff value was 427 ng/ml. Interestingly, although there is a poor correlation between serum fibrinogen and CA19-9 (*r* = 0.28, *p* = 0.04), patients with elevated fibrinogen and CA19-9 (fibrinogen ≥ 1000 ng/ml and CA19-9≥300 U/ml) have a shorter median overall survival (65).

#### 4.1.2 THBS2

Recently, the exploration of proteins secreted from tumor tissues enables a new direction of tumor protein biomarkers. Le et al. identified 696 proteins expressed differently in PDAC tumors and normal pancreatic tissues using nanoLC-Tandem MS analysis. Twenty-one candidate biomarkers secreted from tumor tissues were selected, and subsequently, two proteins selected from plasma samples were independently verified. Among them, thrombospondin‐2 (THBS2) was significantly increased in PDAC tumor tissue and plasma samples (*p* < 0.001, AUC = 0.844), and the combined detection of THBS2 and CA19-9 had high accuracy in the diagnosis of PDAC (AUC = 0.952) (46). THBS2, a biomarker previously considered in patients with PDAC, was also associated with intraductal papillary mucinous neoplasms (IPMN) dysplasia. However, THBS2 itself did not predict IPMN grade (66). Meanwhile, Le’s research revealed the diagnostic potential of THBS2 and CA 19-9 as a combination of diagnostic biomarker, and its clinical value should be explored further.

#### 4.1.3 Fucosylated SERPINA1

Protein glycosylation is crucial for the tumor biological mechanisms, including desmoplasia, malignant transformation, and immune response. Current studies indicate that abnormal glycosylation profiles are considered characteristic of cancer. A recent article has specifically summarized the advances of glycomic biomarkers as biomarkers for PC. Another review article highlights new ideas in PC progression through glycomic and glycoproteomic research. Serpin peptidase inhibitor clade A member 1 (SERPINA1), a protease inhibitor, is essential for biological processes, including angiogenesis, immunoreaction, tumor invasion, and metastasis. Previous studies have reported that strong expression of SERPINA1 is associated with poor prognosis in patients with lung and stomach cancer (67). An iTRAQ-based quantitative glycoproteomics analysis of peptides and glycopeptides was performed using PC plasma (49). Twenty-two peptides were chosen. Among these, the levels of serum SERPINA1 and fucosylated SERPINA1 (fuco-SERPINA1) in PC patients were significantly higher than those in gallstone patients. Fuco-SerpinA1 levels were associated with TNM staging and distant metastasis. Moreover, although fuco-SERPINA1, or SERPINA1, is less able to recognize PC patients than CA 19-9 (AUC: 0.652/0.836 *vs*. 0.914), fuc-Serpina1 may improve CA 19-9’s ability to recognize PC (AUC = 0.956).

#### 4.1.4 Protein Panel

People with diabetes have a much higher risk of PC. Diabetes or hyperglycemia can promote the occurrence and development of PC through a variety of mechanisms. Improving the early screening strategy of PC in patients with diabetes is helpful to the early diagnosis of PC, which improves the prognosis of patients (68). Peng et al. adopted a spectral library-based MS platform to study the role of plasma proteomic changes in the causal relationship between PC and diabetes (47). They established multiple biomarker panels based on 11 selected candidate proteins (including APOA4, CD14, CLEC3B, GSN, HRG, ITIH3, KLKB1, LRG1, SERPINF1, SERPING1, and TIMP1). Cross-validation showed that the diagnostic performance of four panels generated from the 11 proteins was better than that of CA19-19 alone. They were complementary with CA19-9 in the distinction between PDAC diabetes and control. Notably, the addition of CA19-9 enhances the potential of each panel. The accuracy of CA 19-9 combined with biomarker panels in diagnosing PDAC was twice that of CA 19-9 alone. Moreover, urine was also used in the research on protein biomarker panel. One study investigated the urine proteomes to distinguish PDAC from chronic pancreatitis (CP) (48). Biomarkers, including the lymphatic vessel endothelial hyaluronan receptor 1 (LYVE-1), regenerating Protein I Alpha (REG1A), and Trefoil Factor 1 (TFF1), could be useful for detecting early-stage PC.

Overall, CA 19-9 is the only widely used biomarker of PC. Although novel PC biomarkers screened out by MS are not established in clinical routine, the combined detection of novel biomarkers and CA 19-9 is a feasible strategy for diagnosing and predicting PC.

### 4.2 Metabolite Biomarkers

Tumor cells’ anabolic and catabolic pathways have undergone adaptive changes. Metabolic reprogramming has also contributed to the proliferation, metastasis, and invasion of PC cells (69). Hence, exploring the changes of metabolites and metabolic pathways in PC patients is helpful to find new markers related to the occurrence and development of the disease.

#### 4.2.1 Lipid

Lipid metabolism is essential for the progression of PC. In one study, tumor tissues collected for PDAC screening were analyzed by transcriptomics and metabolomics (70). Fifty-five metabolites were identified. Among them, FAs with high connectivity and lipase were significantly reduced in PC. Two of the most representative saturated FAs (palmitate and stearate) were found to have strong inhibitory effects on PC cells. Moreover, they made pioneering use of weighted gene co-expression network analysis (WGCNA) to analyze the metabolic network in PDAC. A total of 157 FA-related genes were identified to be associated with PDAC. Among them, pancreatic lipase is significantly reduced in PDAC and may be a predictor of cancer mortality. Smoking, alcohol, CP, and obesity are major risk factors for PC (71). Shu et al. analyzed the relationship between plasma metabolites and risk factors in 226 patients with PC according to UPLC-Q-TOF-MS (53). They identified 10 metabolites, including tetracosanoic acid, PC (18:1/18:4), coumarin, PC (p-18:0/22:6), PE (22:6/16:0), PS (18:0/18:0), picolinic acid, PC (15:0/18:2), PC (22:5/14:0), and PE (22:6/p-18:1), which were associated with the risk of PC. Specifically, 7 of the 10 metabolites belong to glycerophospholipids, most of which are negatively correlated with the risk of PC. These works indicate that disruption of glycerophospholipids could be a new biomarker panel related to PC.

#### 4.2.2 Amino Acid

IPMN is a rare low-grade malignant pancreatic cystic tumor with slow growth and diverse clinical manifestations. It is frequently misdiagnosed as CP or mucinous cystadenoma. In Section 4.1, the positive role of combined detection of THBS2 and CA 19-9 in diagnosing IPMN was mentioned (46). In addition to protein markers, Nakano and colleagues adopted GC-MS to detect plasma metabolites in PC patients, IPMN patients, and normal volunteers and found that lysine could detect high-risk IPMN, with a sensitivity and specificity of 67.8% and 86.9%, respectively (55).

Researchers have also reported changes in amino acids in PC tissues. Hiraoka et al. adopted LC-MS/MS to map the tissue amino acid profiles from 311 patients with PC. They found that the amino acid profile of PDAC tissue was different from that of normal tissue but similar to that of CP tissue. These findings remind us of the similar urinary headspace VOCs profiles of PDAC and CP (72).

This phenomenon is an interesting connection that researchers can study in-depth in the future. In addition, they also found that some tissue amino acids have characteristic changes during the multi-step carcinogenesis of PC. For instance, during the IPMN progression, Pro, Thr, HyPro, Ile, Asn, Glu, and Tyr remarkably increased. Consequently, tissue amino acid index (TAAI) based on five significantly changed amino acids was established accordingly. Univariate and multivariate survival analysis showed that the survival rate of PDAC patients with high TAAI was substantially lower, suggesting that TAAI can serve as an independent prognostic indicator for PDAC patients (56). Moreover, they did not confirm any correlation between TAAI and serum CA 19-9 and CEA. In the end, there are two points learned from Daulton’s research: (1) researchers should pay attention to the errors caused by the degradation of metabolites in tissue samples; (2) researchers should pay attention to explore the relationship between the changes of amino acids in blood and tissue.

#### 4.2.3 Metabolite Panel

The goal is to explore metabolite changes in patients with advanced unresectable PDAC, Martín et al. adopted RPLC-HRMS to detect the metabolite level in serum samples from 59 unresectable PDAC patients and 60 healthy volunteers (54). They identified 86 metabolites with significant changes in serum samples from PDAC patients. Pathway analysis revealed that four metabolic pathways, namely, linoleic acid metabolism, glycerolipid metabolism, glycerophospholipid metabolism, and primary bile acid biosynthesis, may be linked to unresectable PDAC. In addition, they developed a multivariate model based on nine candidate markers to distinguish unresectable PDAC from HC patients with an AUC value of 0.992. Specifically, low levels of 13-hydroxyoctadecadienoic acid, triglyceride (22:2), 3-oxooctadecanoic acid, oleoyl-L-carnitine, and diacylglycerol CDP-DG (i-24:0/i-17:0), as well as high levels of adrenic acid, lithocholic acid, ethanol chenodeoxycholic acid 7-sulfate, and 4-oxyretinoic acid, can be used as marker metabolites of advanced PDAC (54).

### 4.3 VOC Biomarkers

VOCs, a type of organic compound, exist as steam at ambient temperature. The researchers first discovered that ordinary domestic dogs with basic training could precisely distinguish between breath samples from lung and breast cancer patients and normal subjects’ breath samples (73). Later, many studies investigated the value of VOCs in human cancer (74, 75). To date, studies have revealed that VOCs, as metabolites of the body, can directly reflect the metabolism of body tissues and cells. VOC detection has the advantages of being rapid and non-invasive, which can significantly alleviate the pain of patients undergoing examination. Therefore, VOCs from breath, feces, urine, or sweat have wide-ranging perspectives for new methods of non-invasive tumor screening. This section will specifically discuss the potential of VOCs in alveolar air, urine, and bile in the diagnosis of PC.

#### 4.3.1 Alveolar Air

PC can alter the concentration of specific molecules in the blood, which changes the concentration of VOCs in the alveolar air. Princivalle et al. adopted ion-molecule reaction (IMR)-MS to measure VOCs during breathing at the end of the tide in 75 patients and 144 controls. The results showed that the sensitivity and specificity of the Logistic Regression model based on MS data to map VOC profiles in the alveolar cavity were 100% and 84%, respectively, in the diagnosis of PDAC (50). Ten differential molecules were confirmed, with only five known, namely, ammonia, sulfur dioxide, hydrogen sulfide, acetyl, and acetaldehyde. The chemical identification of the remaining five molecules requires further study.

#### 4.3.2 Urine

Urine testing is one of the easiest, fastest, and most painless screening methods. Previous studies have suggested that the change of microRNA profile in urine is useful for the diagnosis of PC (76). Arasaradnam et al. verified that the variation in urinary VOC profile is a valuable biological fluid, which can distinguish PDAC patients from normal people (AUC = 0.92). They also found that the detection of VOCs can distinguish early-stage PDAC (I/II) from advanced-stage PDAC (III/IV) (AUC = 0.92) (51). Moreover, Daulton et al. identify PDAC and healthy volunteers by analyzing the change of urinary headspace based on GC-IMS or GC-TOF-MS (72). Both GC-IMS data and GC-TOF-MS data are able to distinguish PDAC from healthy controls with good sensitivity and specificity (AUC is more significant than 0.85). 2,6-Dimethyl-octane, nonanal, 4-ethyl-1,2-dimethyl-benzene, and 2-pentanon were essential biomarkers. In addition, the GC-IMS data make a clear distinction between CP and control, while the result of GC-TOF-MS is poor. The important thing is that neither GC-IMS data nor GC-TOF-MS data can distinguish PDAC from CP (both data having an AUC of about 0.7). This result shows that PDAC and CP may produce similar VOCs profiles in the urinary headspace.

#### 4.3.3 Bile

Unlike the VOC profile of urine, Navaneethan et al. found that the VOC profiles in bile based on MS might distinguish PC from CP. The sensitivity and specificity of the logistic model based on bile VOC profile (ammonia, acetonitrile, and trimethylamine) for identifying patients with PC were 93.5% and 100%, respectively (52).

These results show that the unique VOC profile of different body fluids has its advantages in diagnosing PC. Researchers should use more clinical cases to explore further moving forward.

### 4.4 Liquid Biopsy—Exosomes

Exosome is a 30- to 150-nm EV that is released by cells. Exosomes can regulate many biological processes by transferring proteins, lipids, mRNAs, miRNAs, and DNA fragments carried on their own (77). Many studies have highlighted the close relationship between exosomes and tumor angiogenesis, invasion, metastasis, and immune escape (78). It is worth noting that exosomes, which act as a new starting point in the liquid biopsy, may provide a new development direction for early diagnosis and drug resistance evaluation of PC (79).

#### 4.4.1 Exosome Proteins

Exosomal GPC1 is a controversial marker for early diagnosis of PC, discovered for the first time by Melo in 2015 (80). They found that the serum GPC1 concentration was absolutely specific and sensitive in identifying PC patients and normal volunteers. Later, some researchers began to question the accuracy of exosomal GPC1 in PC diagnosis (81). LC-MS/MS analyzes the level of exosomal GPC1 in patients with PDAC. The results indicated that exosomal GPC1 was elevated in patients with PDAC but had little value in the early diagnosis of PDAC (82).

One study used serum from PC patients, pancreatitis patients, and healthy volunteers to examine exosome proteomic profiles. Compared to pancreatitis patients, PC serum exosomes had 29 proteins of differential expression. Compared to the control, PC serum exosomes contained 28 proteins expressed differently. Among them, the authors investigated the upregulated annexin A11 (ANXA11) and hypothesized a link between upregulated protein and the development and process of PC. In comparison with the non-malignant pancreatic epithelial cell lines, ANXA11 was significantly increased in the lysates of four PC cell lines. Interestingly, ANXA11 was only detected in exosomes of the CFPAC-1 cell line. Overall, ANXA11 supports the proliferation and metastasis of PC cells and may serve as a potential biomarker for PC.

#### 4.4.2 Exosomes in Pancreatic Duct Fluid

The PDF is in direct contact with the tumor epithelium and stroma, an ideal environment for the study of PDAC. Zheng et al. used LC-MS/MS to quantitatively analyze the protein composition of PDF exosomes to determine the feasibility of PDF exosome isolation and the clinical significance of measuring exosome proteins. They found that CEACAM1/5 and tenascin C may be potential biomarkers to distinguish benign patients from precancerous lesions (57).

Although exosomal proteins may be used to diagnose PDAC patients, greater attention should be paid to the source of exosomes. Recent studies suggested that EVs secreted from PC cells can initiate the malignant transformation of healthy cells. Servage and colleagues adopted proteomic MS to analyze the difference of proteins in vesicles secreted by normal and cancerous pancreatic cells. They suggested that EVs in PC cells contained unique cargo exosomal proteins, which may be essential for the progression of PC (83). However, the identification of exosomes derived from tumor cells or non-tumor cells in peripheral blood remains a clinical challenge. The recognized exosomal markers CD63, CD9, and CD81, are not tumor-specific. To further determine the function of the PDAC exosome, researchers screened and identified a group of PDAC-specific exosomal surface proteins named “Surfaceome” (molecular marker combinations: LDN4, EPCAM, CD151, LGALS3BP, HIST2H2BE, and HIST2H2BF) (84). These results provide the potential for the clinical application of exosomes. Exosomes should be used as a novel liquid biopsy marker for the early diagnosis of PC.

### 4.5 MS and AI

Recently, AI has become a hot spot in medical research. AI plays an essential role in the diagnosis, treatment, and management of cancer (85). Bile duct stricture is a common clinical condition, which may be divided into benign and malignant bile duct stricture (86). To distinguish whether bile duct stricture is caused by PC, Urman et al. combined metabolomics and proteomics data with AI to distinguish malignant from benign bile duct strictures. Machine learning (ML) methods analyzed the metabolomic and proteomic data obtained from HPLC-MS. Discriminant analysis of principal components (DAPC), random forest (RF), and area under the curve (AUC) were key parameters for evaluating the best lipid combination, including PCs, Cer, DG, and TG species, cholesteryl esters, and phosphatidylinositol. Besides, the dataset was used to train three different ML algorithms: a Bayesian variant of the general linear model (BGLM), C5.0, and neural networks (NN). NN provided the best predictive performance (88% sensitivity and 100% specificity). With a similar approach, the candidate proteins (including MUC5B, FAT4, ALB, AMY2A, and ENPP7) identified by DAPC analysis can get 100% sensitivity and 100% specificity using NN (87). These findings support the potential of ML and MS analysis in selecting PC biomarkers.

Moreover, Chung and his colleagues have developed a unique diagnosis system for PC on MS and AI. They adopted the PESI-MS system to analyze serum samples from patients with PDAC and normal volunteers and then entered the MS of each sample into an ML algorithm for analysis. More than 90% of PDAC cases and normal control cases could be distinguished based on the PESI-MS and ML data analyzed by the diagnostic system, with a sensitivity and specificity of 91.7% and 90.4% (88), outperforming CA19-9 for the diagnosis of PDAC. Anyway, the combination of AI and MS is helpful to improve the early diagnosis rate and accuracy of PC.

In a word, novel protein or metabolite biomarkers have been identified from MS, and the links between biomarkers and PC progression were analyzed by MS-based studies. The advantages of MS are apparent. The emergence of MS has made more sample types possible as biomarkers for clinical research. Moreover, high-throughput MS data can be combined with artificial data to significantly accelerate the discovery of biomarkers.

## 5 MS and Treatment of PC

Radical resection is the most effective method for the treatment of PC. However, most patients with PC have lost the opportunity for surgery when they are diagnosed. With the development of local therapy, targeted therapy, and immunotherapy, various treatments with a comprehensive treatment of PC as the core have shown obvious advantages (89, 90). Fortunately, the MS application can help solve the “general problem” of comprehensive therapy for PC.

### 5.1 Gemcitabine

Pharmacotherapy with GEM is the essential treatment for advanced PDAC (91). Previous studies have suggested that GEM’s efficacy depends on the tissue transmission and distribution of the drug within the pancreas. However, the commonly used autoradiography and tissue homogenate LC-MS still have significant limitations in providing a comprehensive evaluation of tissue distributions (92). Grüner et al. developed a MALDI-MIS-based detection approach to analyze the delivery and distribution of GEM in normal and cancerous pancreatic tissue (93). MALDI drug imaging is a targeting method used to detect specific drugs in samples, which can effectively distinguish parent drugs and metabolites without changing the spatial distribution of the drug in tissues (94). Grüner et al. showed that the GEM levels in PDAC tissue were significantly lower than that in normal pancreatic tissue, and the PDAC mice with a higher peak intensity of GEM in the pancreas showed a higher survival rate. In general, MALDI drug imaging provides an important direction for the transformation of chemotherapeutic drugs in preclinical and clinical trials (93). Resistance to chemotherapy is a crucial challenge for GEM in the treatment of PC. To better predict the response to chemotherapy and prevent drug resistance, researchers explored potential biomarkers to predict the effectiveness of PC chemotherapy (95). Wu et al. adopted UPLC/Q-TOFMS for mapping the metabolic profiles following GEM monotherapy or combination therapy. They found that both monotherapy and combination therapy contribute to significant metabolic changes, and the relative levels of 3-hydroxyadipic acid, d-galactose, LysoPC (P-16:0), and tetradecenoyl-l-carnitine could be regarded as biomarkers to predict the efficacy of chemotherapy (58).

Interestingly, recent studies have pointed out that leflunomide (Lef) synergizes with GEM in inhibiting PC cell growth. Lef is an immunosuppressive agent that reduces *de novo* synthesis of pyrimidines by inhibiting dihydrowhey acid dehydrogenase (DHODH). By experiments *in vitro*, Teriflunomide (the metabolically active ingredient of Lef) inhibited PC cell growth by inhibiting DHODH and PIM-3 kinase and synergized with GEM (96). Based on previous research, Phan et al. mapped the changes in metabolites of PC cells after combination treatment (97). Lef has been shown to increase the sensitivity of PC cells to GEM by inhibiting the *de novo* pyrimidine synthesis. Specifically, the combination therapy inhibited PC cell proliferation and angiogenesis by inhibiting the expression of DHODH and induced favorable anti-tumor immune phenotypes. In conclusion, metabolomics suggests that *de novo* pyrimidine synthesis may be a potential mechanism for reducing PC resistance. The combined Lef and GEM therapy may be a focus of study for patients with PC.

### 5.2 Immunotherapy

Immunotherapy has become a hot spot in PC research, and this is also the main development direction in the future. Immunotherapy, including programmed death protein-1/programmed death protein ligand-1 (PD-1/PD-L1) antibody, and cytotoxic T lymphocyte antigen-4 (CTLA-4) antibody, was extensively studied (98). Recent studies have revealed that a variety of PC-specific membrane proteins can also help the immune escape of tumor cells. Based on MS analysis, Meng et al. found a novel panel of PC-specific membrane proteins (including CD36, EPB42, CASK, etc.) that can be used as therapeutic targets for PC (99). Immune checkpoints, a kind of inhibitory signal pathway, help tumor cells escape from the monitoring and recognition of the immune system (100). Ideally, checkpoint inhibitors (CPI) exert anti-tumor effects by non-specific activation of T lymphocytes. However, studies have shown that the efficacy of immunotherapy based on CPI and CTLA-4 in PC treatment was not expected to be effective (101). Bradley and colleagues analyzed the tumor-associated peptides derived from 35 PDAC samples according to tandem MS (59). They found that the Vestigial-like 1 expression was inversely correlated with the survival of patients with PDAC. Vestigial-like 1, a specific marker of proliferative and invasive cytotrophoblast cells that is expressed in several cancers, can become a safe and novel tumor-associated antigen (TAA) for PC.

Stromal cells, including fibroblasts, T cells, mesenchymal cells, and PCSCs, are critical in the metastasis and invasion of PC. Several review papers suggested that targeting stromal cell populations can enhance the anti-tumor immune response of PC (102, 103). Some scholars believe that the complement pathway in stromal elements could support tumor proliferation through immunological tumor promotion. Complement factor B (CFB) is essential to complement alternative pathways. CFB has previously been reported as a serum biomarker for PC (104). Another study suggested that plasma CFB was superior to CA 19-9 and CEA as a prognostic biomarker for resectable PC (105). Recently, high stromal CFB expression in the PDAC samples was determined by HPLC-MS/MS analysis (106). Silencing of CFB could significantly inhibit PC cell proliferation and promote senescence in PC. Indeed, high CFB expression was also associated with hematogenetic recurrence and poor prognosis of PDAC. More importantly, CFB was associated with increased expression of CD8^+^/Foxp3^+^ Tregs in the tumor microenvironment. Accumulation of the latter may promote immune escape. Therefore, CFB is a potential therapeutic target for immunomodulatory therapy in PC.

### 5.3 Targeted Therapy

Targeted molecular therapy is seen as the gateway to hope for PC treatment due to its specific and effective lethal effect on cancer cells. Currently, targeted therapy for PC is primarily designed to inhibit molecular pathways and alter metabolic pathways. The ongoing development of MS has provided new hope for accurately targeted therapy of PC.

Signal transduction induced by protein kinase phosphorylation is an important pathway to affect the progression of PDAC. The phosphoproteome analysis by Le and his colleagues confirmed the role of focal adhesion kinase (FAK) as a new target for PDAC. FAK was the core participant of hyperphosphorylated tyrosine kinases in PDAC, while the combination of FAK inhibitors with paclitaxel showed a synergistic anti-tumor effect on PDAC cells *in vitro* and *in vivo* (107). Besides protein phosphorylation, abnormal ubiquitin also plays an essential role in cancer metastasis. UBR5, a ubiquitin ligase, is upregulated in many cancer samples, such as colorectal and gastric cancers and associated with tumor growth. Recently, studies have found that UBR5 was increased in PC, and is associated with a poor prognosis, targeting UBR5 or UBR5-mediated CAPZA1 ubiquitination may be a promising strategy for patients with PC (108). To better elucidate the role of UBR5 in PC cells, they identified CAPZA1 as a new substrate of UBR5 by co-immunoprecipitation combined with MS analyses. UBR5 can promote cancer cell migration and invasion by promoting CAPZA1 deficiency and inducing F-actin accumulation.

Metabolic deregulation is the vital way for the occurrence and development of tumors, in which changes in amino acid metabolism are closely associated with the phenotype of cancer. Metabolic abnormalities of the branched-chain amino acid (BCAA), including leucine isoleucine and valine, have been reported to promote the metabolism of various malignant tumors, including hepatocellular carcinoma and leukemia. Similarly, Jiang et al. adopted nano-LC-MS/MS to determine protein expression in PDAC and adjacent tissues, and KEGG enrichment analysis highlighted the importance of BCAA in PDAC. Moreover, significantly upregulated BCAA was observed in clinical samples of PDAC by NMR determination. However, BCAA had no influence on the proliferation or migration of PDAC cells *in vitro* experiments. It was speculated that BCAAs in a typical cell culture environment were significantly higher than the physiological level. Finally, the regulation of BCAA metabolism in PDAC by pancreatic stellate cells gives us a meaningful direction (109). It is worth noting that Ke et al. found that BCAA encourages PC cell proliferation and inhibits the cell apoptosis induced by oxaliplatin *in vitro*. They used endoscopic ultrasound-guided fine-needle aspiration (EUS-FNA) samples for metabolomic analysis. The results suggest that BCAA biosynthesis, glycerol metabolism, and phenylalanine metabolism are the top three main metabolic pathways that affect the pancreatic head and body/tail adenocarcinoma. Thus, anti-BCAA metabolic therapy may be a promising therapeutic target for the above two types of PC (110).

In addition to BCAA, lysophosphatidic acid (LPA) can activate several downstream signaling cascades by binding to specific receptors and participating in the PC process. LPA, a lipid signal, promotes cancer cell migration and development. To explore the potential mechanism of the effect of LPA on PC, Shi et al. used co-immunoprecipitation in combination with MS analyses to evaluate the role of CMTM8 as an LPA1-associated target in the migration and invasion of PC cells. The results showed that β-catenin activation is an important event downstream of LPA1 (LPA receptor)—CMTM8, PRDX2, CAP1, ALKBH5, and RACK1 relate to LPA1 through MS analysis of LPA1 immunoprecipitate (111).

### 5.4 Others

Arsenic trioxide (ATO), a mineral drug approved by the U.S. Food and Drug Administration (FDA), is widely used to treat acute promyelocytic leukemia. The researchers discovered that ATO can induce apoptosis of PC cells by disrupting the ubiquitin–proteasome pathway. To expand the anti-cancer effect of ATO, they tried to combine pyrrolidine dithiocarbamate (PDTC) with ATO to form a stable complex (to prevent arsenic ions from being trapped by detoxifying compounds) to play an anti-cancer effect. SFC-MS data showed that PDTC and ATO could form a stable complex; the formed PDTC–ATO complex could inhibit the activity of PC cells by interfering with the ubiquitin–proteasome pathway, which showed better activities than ATO (112). Radiofrequency ablation (RFA), which is part of the local therapy, is currently the most widely used tumor ablation therapy (113). With the development of RFA equipment and treatment technology, researchers have begun to explore the potential of RFA in the treatment of locally advanced PC. Gao et al. identified the differentially expressed proteins (DEPs) related to RFA by MS; they found that heat shock protein 70 was the highest DEP. Heat shock protein 70 could promote cell proliferation by activating the AKT–mTOR signaling pathway. Subsequently, an increase in tumor immune response and inhibition of residual tumor cell proliferation was observed in the PC model treated with the combination therapy of RFA with mTOR inhibitors (114).

Betulinic acid (BA) is a pentacyclic triterpene with potential anti-tumor effects on many cancers, including colorectal cancer, lung cancer, and PC. BA shows selective antitumor activity, which could significantly inhibit the proliferation, metastasis, and invasion of tumor cells without harming normal cells (115). Previous studies have shown that the antitumor effect of BA relates to the mitochondrial pathway-mediated oxidative damage, but the specific mechanism has not been clarified. Chiu and colleagues identified the potential mechanism of BA regulating mitochondrial energy metabolism within PDAC cells through proteomics (116). As with previous reports, BA significantly inhibited PDAC cell growth without affecting normal cells. Compared to PDAC, seven clusters containing 3,316 DEPs were regulated by BA. Three of the clusters were then identified for SAM analysis and functional enrichment analysis. The DEPs NADH:ubiquinone oxidoreductase core subunit S2 (NDUFS2), RNA polymerase mitochondrial (POLRMT), and translational activator of cytochrome c oxidase (TACO1) are mitochondrial function-related proteins. Among them, the high expression of NDUFS2 promotes mitochondrial activity and ATP production, which is conducive to the proliferation and invasion of PC cells; POLRMT plays a key role in the initiation of mitochondrial genome transcription and can promote the metabolism and growth of tumor cells; TACO1 is a translation-activating protein that promotes oxidative phosphorylation in tumor cells. As expected, BA can inhibit the expression of these proteins and thus play an anti-tumor role in PC. In sum, these findings highlight the potential pharmacological effect of BA against PC, which highlights the power of proteomics in determining the anti-tumor properties of drugs.

Overall, we showed recent main findings regarding advances in MS-mediated PC therapies. MALDI-MIS or LC-MS could be combined with current treatments to improve the prognosis of patients with PC.

## 6 Conclusion and Perspectives

In a word, MS plays a significant role in the translational research of PC: (1) MS helps researchers explain the relationship between risk factors and PC pathogenesis. (2) MS helps to mine biomarkers in serum, bile, urine, and PDF; in particular, AI that aids in analyzing MS data is also exciting. (3) In terms of treatment, MS technology is useful to monitor the efficacy of chemotherapy and screen the targets of immunotherapy and targeted therapy. The research mentioned above has shown that MS has an extensive perspective in cancer research. The combination of MS and systems biology (including transcriptomics, proteomics, and metabolomics), ML, and bioinformatics brings new hope for precision medicine and personalized treatment of tumors. Specifically, (1) the new ionization methods (PESI and MALDI) provide an essential platform for clinical research of cancer, and proteomics based on MS can screen, diagnose, and detect cancer prognosis. (2) GC-MS and LC-MS are the best platforms for detecting metabolite markers, which have broad perspectives in the pathogenesis and diagnosis of cancer. (3) MSI technology adds new vitality to the pathological analysis of cancer and may promote the personalized treatment of cancer patients. Therefore, MS is a significant developmental direction for the clinical laboratory in the future.

However, there are still some urgent problems to be solved for the further development and application of MS in PC: (1) Given the issues of sample size, analysis platform, and laboratory conditions, several new biomarkers discussed in this review need to be further verified in extensive cohort clinical studies in order to evaluate their potential clinical efficacy. Notably, a group of biomarkers is gradually changing the diagnostic value of a single marker for PC. (2) The data and information obtained from MS are highly complex, and require comprehensive biological and functional annotation tools. A more stable bridge must be constructed by a wide range of scientists. (3) MALDI-MSI brings new impetus to the field of spatial analysis of tumor tissue sections, and limited spatial resolution should be enhanced for its clinical application. In any event, the application of MS in PC will continue to increase along with the solution of the above problems based on the continuous improvement of methods and equipment.

## Author Contributions

HLC provided manuscript design. PG, YL, HYC, JL, HG, CX, JQ, and GZ drafted the manuscript. All authors contributed to manuscript revision, read, and approved the submitted version.

## Funding

This study was supported by the key project of the National significant R&D Program “Intergovernmental Cooperation in International Science and Technology Innovation” (grant number 2019YFE0119300) and the National Natural Science Foundation of China (grant number 82074158).

## Conflict of Interest

The authors declare that the research was conducted in the absence of any commercial or financial relationships that could be construed as a potential conflict of interest.

## Publisher’s Note

All claims expressed in this article are solely those of the authors and do not necessarily represent those of their affiliated organizations, or those of the publisher, the editors and the reviewers. Any product that may be evaluated in this article, or claim that may be made by its manufacturer, is not guaranteed or endorsed by the publisher.

## Abbreviations

PC, pancreatic cancer; MS, mass spectrometry; PDAC, pancreatic ductal adenocarcinoma; LC, liquid chromatography; GC, gas chromatography; VOCs, volatile organic compounds; MALDI, matrix-assisted laser desorption ionization; MSI, mass spectrometry imaging; MUC4, Mucin-4; CA19-9, Carbohydrate antigen 19-9; AI, artificial intelligence; THBS2, Thrombospondin‐2; CP, chronic pancreatitis; IPMN, intraductal papillary mucinous neoplasms; TAAI, tissue amino acid index; PDF, pancreatic duct fluid; ML, machine learning; NN, neural networks; GEM, Gemcitabine; BCAA, branched-chain amino acid; LPA, lysophosphatidic acid; ATO, arsenic trioxide; EVs, extracellular vesicles; PCSCs, pancreatic cancer stem cells; FAs, fatty acids; SERPINA1, serpin peptidase inhibitor clade A member 1; DHODH, dihydrowhey acid dehydrogenase; Lef, leflunomideb; BA, betulinic acid; WGCNA, weighted gene co-expression network analysis; ANXA11, annexin A11; PCa, prostate cancer; FFPE, formalin-fixed, paraffin-embedded.

## References

[1] Sipra Q Islam M Riaz IB Zhaohui J Babiker HM Bekaii-Saab TS. Contemporary Management of Pancreatic Cancer From an Internist Perspective. Am J Med (2020) 134(5):576–6. doi: 10.1016/j.amjmed.2020.11.009 33316248

[2] Turpin A El Amrani M Bachet JB Pietrasz D Schwarz L Hammel P. Adjuvant Pancreatic Cancer Management: Towards New Perspectives in 2021. Cancers (Basel) (2020) 12(12):3866. doi: 10.3390/cancers12123866 PMC7767489 33371464

[3] Bengtsson A Andersson R Rahm J Ganganna K Andersson B Ansari D. Organoid Technology for Personalized Pancreatic Cancer Therapy. Cell Oncol (Dordr) (2021) 44(2):251–60. doi: 10.1007/s13402-021-00585-1 PMC7985124 33492660

[4] McGuigan AJ Coleman HG McCain RS Kelly AJ Johnston HG Taylor RS. Immune Cell Infiltrates as Prognostic Biomarkers in Pancreatic Ductal Adenocarcinoma: A Systematic Review and Meta-Analysis. J Pathol Clin Res (2021) 7(2):99–112. doi: 10.1002/cjp2.192 33481339 PMC7869931

[5] Siegel RL Miller KD Fuchs HE Jemal A. Cancer Statistics, 2021. CA Cancer J Clin (2021) 71(1):7–33. doi: 10.3322/caac.21654 33433946

[6] Austin CP. Translating Translation. Nat Rev Drug Discovery (2018) 17(7):455–6. doi: 10.1038/nrd.2018.27 PMC6023744 29674698

[7] Chong YK Ho CC Leung SY Lau S Woo P. Clinical Mass Spectrometry in the Bioinformatics Era: A Hitchhiker’s Guide. Comput Struct Biotechnol J (2018) 16:316–34. doi: 10.1016/j.csbj.2018.08.003 PMC6138949 30237866

[8] Fung A Sugumar V Ren AH Kulasingam V. Emerging Role of Clinical Mass Spectrometry in Pathology. J Clin Pathol (2020) 73(2):61–9. doi: 10.1136/jclinpath-2019-206269 31690564

[9] Tholl D Hossain O Weinhold A Röse U Wei Q. Trends and Applications in Plant Volatile Sampling and Analysis. Plant J (2021) 106(2):314–25. doi: 10.1111/tpj.15176 33506558

[10] Bannaga AS Tyagi H Daulton E Covington JA Arasaradnam RP. Exploratory Study Using Urinary Volatile Organic Compounds for the Detection of Hepatocellular Carcinoma. Molecules (2021) 26(9):2447. doi: 10.3390/molecules26092447 33922256 PMC8122735

[11] Woollam M Wang L Grocki P Liu S Siegel AP Kalra M. Tracking the Progression of Triple Negative Mammary Tumors Over Time by Chemometric Analysis of Urinary Volatile Organic Compounds. Cancers (Basel) (2021) 13(6):1462. doi: 10.3390/cancers13061462 33806757 PMC8004946

[12] Wang W He Z Kong Y Liu Z Gong L. GC-MS-Based Metabolomics Reveals New Biomarkers to Assist the Differentiation of Prostate Cancer and Benign Prostatic Hyperplasia. Clin Chim Acta (2021) 519:10–7. doi: 10.1016/j.cca.2021.03.021 33831421

[13] Tan B Zhang Y Zhang T He J Luo X Bian X. Identifying Potential Serum Biomarkers of Breast Cancer Through Targeted Free Fatty Acid Profiles Screening Based on a GC-MS Platform. BioMed Chromatogr (2020) 34:e4922. doi: 10.1002/bmc.4922 32537761

[14] Wojakowska A Marczak Ł Jelonek K Polanski K Widlak P Pietrowska M. An Optimized Method of Metabolite Extraction From Formalin-Fixed Paraffin-Embedded Tissue for GC/MS Analysis. PloS One (2015) 10:e0136902. doi: 10.1371/journal.pone.0136902 26348873 PMC4562636

[15] Buszewska-Forajta M Raczak-Gutknecht J Artymowicz M Wesołowski W Buczkowski K Iżycka-Świeszewska E. The Potential Role of Fatty Acids in Prostate Cancer Determined by GC-MS Analysis of Formalin-Fixed Paraffin-Embedded Tissue Samples. J Pharm BioMed Anal (2021) 196:113907. doi: 10.1016/j.jpba.2021.113907 33497978

[16] Andries A Rozenski J Vermeersch P Mekahli D Van Schepdael A. Recent Progress in the LC-MS/MS Analysis of Oxidative Stress Biomarkers. Electrophoresis (2020) 42(4):402–28. doi: 10.1002/elps.202000208 33280143

[17] Segers K Declerck S Mangelings D Heyden YV Eeckhaut AV. Analytical Techniques for Metabolomic Studies: A Review. Bioanalysis (2019) 11:2297–318. doi: 10.4155/bio-2019-0014 31845604

[18] Zeki ÖC Eylem CC Reçber T Kır S Nemutlu E. Integration of GC-MS and LC-MS for Untargeted Metabolomics Profiling. J Pharm BioMed Anal (2020) 190:113509. doi: 10.1016/j.jpba.2020.113509 32814263

[19] Vinaiphat A Low JK Yeoh KW Chng WJ Sze SK. Application of Advanced Mass Spectrometry-Based Proteomics to Study Hypoxia Driven Cancer Progression. Front Oncol (2021) 11:559822. doi: 10.3389/fonc.2021.559822 33708620 PMC7940826

[20] Momenbeitollahi N Cloet T Li H. Pushing the Detection Limits: Strategies Towards Highly Sensitive Optical-Based Protein Detection. Anal Bioanal Chem (2021) 413(24):5995–6011. doi: 10.1007/s00216-021-03566-3 34363087 PMC8346249

[21] Florio W Baldeschi L Rizzato C Tavanti A Ghelardi E Lupetti A. Detection of Antibiotic-Resistance by MALDI-TOF Mass Spectrometry: An Expanding Area. Front Cell Infect Microbiol (2020) 10:572909. doi: 10.3389/fcimb.2020.572909 33262954 PMC7686347

[22] Cui JJ Wang LY Tan ZR Zhou HH Zhan X Yin JY. MASS SPECTROMETRY-BASED PERSONALIZED DRUG THERAPY. Mass Spectrom Rev (2020) 39:523–52. doi: 10.1002/mas.21620 31904155

[23] Paglia G Smith AJ Astarita G. Ion Mobility Mass Spectrometry in the Omics Era: Challenges and Opportunities for Metabolomics and Lipidomics. Mass Spectrom Rev (2021) 2021:1–44. doi: 10.1002/mas.21686 33522625

[24] Witt B Schaumlöffel D Schwerdtle T. Subcellular Localization of Copper-Cellular Bioimaging With Focus on Neurological Disorders. Int J Mol Sci (2020) 21(7):2341. doi: 10.3390/ijms21072341 PMC7178132 32231018

[25] Nassar SF Raddassi K Ubhi B Doktorski J Abulaban A. Precision Medicine: Steps Along the Road to Combat Human Cancer. Cells (2020) 9(9):2056. doi: 10.3390/cells9092056 PMC7563722 32916938

[26] Abdel Mouti M Pauklin S. TGFB1/INHBA Homodimer/Nodal-SMAD2/3 Signaling Network: A Pivotal Molecular Target in PDAC Treatment. Mol Ther (2021) 29(3):920–36. doi: 10.1016/j.ymthe.2021.01.002 PMC7934636 33429081

[27] Khan AA Liu X Yan X Tahir M Ali S Huang H. An Overview of Genetic Mutations and Epigenetic Signatures in the Course of Pancreatic Cancer Progression. Cancer Metastasis Rev (2021) 40(1):245–72. doi: 10.1007/s10555-020-09952-0 33423164

[28] Tao J Yang G Zhou W Qiu J Chen G Luo W. Targeting Hypoxic Tumor Microenvironment in Pancreatic Cancer. J Hematol Oncol (2021) 14(1):14. doi: 10.1186/s13045-020-01030-w 33436044 PMC7805044

[29] Gautam SK Kumar S Dam V Ghersi D Jain M Batra SK. MUCIN-4 (MUC4) Is a Novel Tumor Antigen in Pancreatic Cancer Immunotherapy. Semin Immunol (2020) 47:101391. doi: 10.1016/j.smim.2020.101391 31952903 PMC7160012

[30] Gál E Veréb Z Kemény L Rakk D Szekeres A Becskeházi E. Bile Accelerates Carcinogenic Processes in Pancreatic Ductal Adenocarcinoma Cells Through the Overexpression of MUC4. Sci Rep (2020) 10(1):22088. doi: 10.1038/s41598-020-79181-6 33328627 PMC7744548

[31] Scheufele F Schorn S Demir IE Sargut M Tieftrunk E Calavrezos L. Preoperative Biliary Stenting *Versus* Operation First in Jaundiced Patients Due to Malignant Lesions in the Pancreatic Head: A Meta-Analysis of Current Literature. Surgery (2017) 161(4):939–50. doi: 10.1016/j.surg.2016.11.001 28043693

[32] Duvillié B Kourdoughli R Druillennec S Eychène A Pouponnot C. Interplay Between Diabetes and Pancreatic Ductal Adenocarcinoma and Insulinoma: The Role of Aging, Genetic Factors, and Obesity. Front Endocrinol (Lausanne) (2020) 11:563267. doi: 10.3389/fendo.2020.563267 33101198 PMC7556217

[33] Pergolini I Schorn S Jäger C Göß R Novotny A Friess H. Diabetes Mellitus in Intraductal Papillary Mucinous Neoplasms: A Systematic Review and Meta-Analysis. Surgery (2021) 169(2):411–8. doi: 10.1016/j.surg.2020.07.006 32838986

[34] Velazquez-Torres G Fuentes-Mattei E Choi HH Yeung SJ Meng X Lee MH. Diabetes Mellitus Type 2 Drives Metabolic Reprogramming to Promote Pancreatic Cancer Growth. Gastroenterol Rep (Oxf) (2020) 8(4):261–76. doi: 10.1093/gastro/goaa018 PMC7434590 32843973

[35] Guo H Deng H Liu H Jian Z Cui H Fang J. Nickel Carcinogenesis Mechanism: Cell Cycle Dysregulation. Environ Sci Pollut Res Int (2021) 28(5):4893–901. doi: 10.1007/s11356-020-11764-2 33230792

[36] Bjørklund G Pivina L Dadar M Semenova Y Chirumbolo S Aaseth J. Mercury Exposure, Epigenetic Alterations and Brain Tumorigenesis: A Possible Relationship. Curr Med Chem (2020) 27(39):6596–610. doi: 10.2174/0929867326666190930150159 31566127

[37] Kim H Lee J Woo HD Kim DW Oh JH Chang HJ. Dietary Mercury Intake and Colorectal Cancer Risk: A Case-Control Study. Clin Nutr (2020) 39:2106–13. doi: 10.1016/j.clnu.2019.08.025 31522783

[38] Pamphlett R Colebatch AJ Doble PA Bishop DP. Mercury in Pancreatic Cells of People With and Without Pancreatic Cancer. Int J Environ Res Public Health (2020) 17(23):8990. doi: 10.3390/ijerph17238990 PMC7731371 33276658

[39] Lenart M Rutkowska-Zapala M Baj-Krzyworzeka M Szatanek R Węglarczyk K Smallie T. Hyaluronan Carried by Tumor-Derived Microvesicles Induces IL-10 Production in Classical (CD14++CD16-) Monocytes *via* PI3K/Akt/mTOR-Dependent Signalling Pathway. Immunobiology (2017) 222:1–10. doi: 10.1016/j.imbio.2015.06.019 26210045

[40] Nannan L Oudart JB Monboisse JC Ramont L Brassart-Pasco S Brassart B. Extracellular Vesicle-Dependent Cross-Talk in Cancer-Focus on Pancreatic Cancer. Front Oncol (2020) 10:1456. doi: 10.3389/fonc.2020.01456 32974169 PMC7466446

[41] Hayasaka R Tabata S Hasebe M Ikeda S Ohnuma S Mori M. Metabolomic Analysis of Small Extracellular Vesicles Derived From Pancreatic Cancer Cells Cultured Under Normoxia and Hypoxia. Metabolites (2021) 11(4):215. doi: 10.3390/metabo11040215 33915936 PMC8066639

[42] Tabang DN Ford M Li L. Recent Advances in Mass Spectrometry-Based Glycomic and Glycoproteomic Studies of Pancreatic Diseases. Front Chem (2021) 9:707387. doi: 10.3389/fchem.2021.707387 34368082 PMC8342852

[43] Di Carlo C Sousa BC Manfredi M Brandi J Dalla Pozza E Marengo E. Integrated Lipidomics and Proteomics Reveal Cardiolipin Alterations, Upregulation of HADHA and Long Chain Fatty Acids in Pancreatic Cancer Stem Cells. Sci Rep (2021) 11:13297. doi: 10.1038/s41598-021-92752-5 34168259 PMC8225828

[44] Khomiak A Brunner M Kordes M Lindblad S Miksch RC Öhlund D. Recent Discoveries of Diagnostic, Prognostic and Predictive Biomarkers for Pancreatic Cancer. Cancers (Basel) (2020) 12(11):3234. doi: 10.3390/cancers12113234 PMC7692691 33147766

[45] Zhong A Qin R Qin W Han J Gu Y Zhou L. Diagnostic Significance of Serum IgG Galactosylation in CA19-9-Negative Pancreatic Carcinoma Patients. Front Oncol (2019) 9:114. doi: 10.3389/fonc.2019.00114 30873386 PMC6402387

[46] Le Large T Meijer LL Paleckyte R Boyd LNC Kok B Wurdinger T. Combined Expression of Plasma Thrombospondin-2 and CA19-9 for Diagnosis of Pancreatic Cancer and Distal Cholangiocarcinoma: A Proteome Approach. Oncologist (2020) 25(4):e634–43. doi: 10.1634/theoncologist.2019-0680 PMC7160420 31943574

[47] Peng H Pan S Yan Y Brand RE Petersen GM Chari ST. Systemic Proteome Alterations Linked to Early Stage Pancreatic Cancer in Diabetic Patients. Cancers (Basel) (2020) 12(6):1534. doi: 10.3390/cancers12061534 PMC7352938 32545216

[48] Radon TP Massat NJ Jones R Alrawashdeh W Dumartin L Ennis D. Identification of a Three-Biomarker Panel in Urine for Early Detection of Pancreatic Adenocarcinoma. Clin Cancer Res (2015) 21:3512–21. doi: 10.1158/1078-0432.CCR-14-2467 PMC4539580 26240291

[49] Wu CC Lu YT Yeh TS Chan YH Dash S Yu JS. Identification of Fucosylated SERPINA1 as a Novel Plasma Marker for Pancreatic Cancer Using Lectin Affinity Capture Coupled With iTRAQ-Based Quantitative Glycoproteomics. Int J Mol Sci (2021) 22(11):6079. doi: 10.3390/ijms22116079 34199928 PMC8200073

[50] Princivalle A Monasta L Butturini G Bassi C Perbellini L. Pancreatic Ductal Adenocarcinoma Can be Detected by Analysis of Volatile Organic Compounds (VOCs) in Alveolar Air. BMC Cancer (2018) 18(1):529. doi: 10.1186/s12885-018-4452-0 29728093 PMC5935919

[51] Arasaradnam RP Wicaksono A O’Brien H Kocher HM Covington JA Crnogorac-Jurcevic T. Noninvasive Diagnosis of Pancreatic Cancer Through Detection of Volatile Organic Compounds in Urine. Gastroenterology (2018) 154(3):485–487.e1. doi: 10.1053/j.gastro.2017.09.054 29129714

[52] Navaneethan U Spencer C Zhu X Vargo JJ Grove D Dweik RA. Volatile Organic Compounds in Bile Can Distinguish Pancreatic Cancer From Chronic Pancreatitis: A Prospective Observational Study. Endoscopy (2020) 53(7):732–36. doi: 10.1055/a-1255-9169 32894868

[53] Shu X Zheng W Yu D Li HL Lan Q Yang G. Prospective Metabolomics Study Identifies Potential Novel Blood Metabolites Associated With Pancreatic Cancer Risk. Int J Cancer (2018) 143(9):2161–7. doi: 10.1002/ijc.31574 PMC6195470 29717485

[54] Martín-Blázquez A Jiménez-Luna C Díaz C Martínez-Galán J Prados J Vicente F. Discovery of Pancreatic Adenocarcinoma Biomarkers by Untargeted Metabolomics. Cancers (Basel) (2020) 12(4):1002. doi: 10.3390/cancers12041002 PMC7225994 32325731

[55] Nakano R Nishiumi S Kobayashi T Ikegawa T Kodama Y Yoshida M. Possibility of Detecting Intraductal Papillary Mucinous Neoplasms Using Metabolite Biomarkers for Pancreatic Cancer. Biomark Med (2020) 14(11):1009–20. doi: 10.2217/bmm-2019-0587 32940075

[56] Hiraoka N Toue S Okamoto C Kikuchi S Ino Y Yamazaki-Itoh R. Tissue Amino Acid Profiles Are Characteristic of Tumor Type, Malignant Phenotype, and Tumor Progression in Pancreatic Tumors. Sci Rep (2019) 9(1):9816. doi: 10.1038/s41598-019-46404-4 31285536 PMC6614459

[57] Zheng J Hernandez JM Doussot A Bojmar L Zambirinis CP Costa-Silva B. Extracellular Matrix Proteins and Carcinoembryonic Antigen-Related Cell Adhesion Molecules Characterize Pancreatic Duct Fluid Exosomes in Patients With Pancreatic Cancer. HPB (Oxford) (2018) 20(7):597–604. doi: 10.1016/j.hpb.2017.12.010 29339034 PMC6779041

[58] Wu D Li X Zhang X Han F Lu X Liu L. Pharmacometabolomics Identifies 3-Hydroxyadipic Acid, D-Galactose, Lysophosphatidylcholine (P-16:0), and Tetradecenoyl-L-Carnitine as Potential Predictive Indicators of Gemcitabine Efficacy in Pancreatic Cancer Patients. Front Oncol (2019) 9:1524. doi: 10.3389/fonc.2019.01524 32064236 PMC7000527

[59] Bradley SD Talukder AH Lai I Davis R Alvarez H Tiriac H. Vestigial-Like 1 Is a Shared Targetable Cancer-Placenta Antigen Expressed by Pancreatic and Basal-Like Breast Cancers. Nat Commun (2020) 11(1):5332. doi: 10.1038/s41467-020-19141-w 33087697 PMC7577998

[60] Lee T Teng T Shelat VG. Carbohydrate Antigen 19-9 - Tumor Marker: Past, Present, and Future. World J Gastrointest Surg (2020) 12:468–90. doi: 10.4240/wjgs.v12.i12.468 PMC7769746 33437400

[61] Goonetilleke KS Siriwardena AK. Systematic Review of Carbohydrate Antigen (CA 19-9) as a Biochemical Marker in the Diagnosis of Pancreatic Cancer. Eur J Surg Oncol (2007) 33:266–70. doi: 10.1016/j.ejso.2006.10.004 17097848

[62] Ballehaninna UK Chamberlain RS. The Clinical Utility of Serum CA 19-9 in the Diagnosis, Prognosis and Management of Pancreatic Adenocarcinoma: An Evidence Based Appraisal. J Gastrointest Oncol (2012) 3:105–19. doi: 10.3978/j.issn.2078-6891.2011.021 PMC3397644 22811878

[63] Fahrmann JF Schmidt CM Mao X Irajizad E Loftus M Zhang J. Lead-Time Trajectory of CA19-9 as an Anchor Marker for Pancreatic Cancer Early Detection. Gastroenterology (2021) 160:1373–83.e6. doi: 10.1053/j.gastro.2020.11.052 33333055 PMC8783758

[64] Ren S Zhang Z Xu C Guo L Lu R Sun Y. Distribution of IgG Galactosylation as a Promising Biomarker for Cancer Screening in Multiple Cancer Types. Cell Res (2016) 26:963–6. doi: 10.1038/cr.2016.83 PMC4973333 27364686

[65] Chung KH Lee JC Lee J Cho IK Kim J Jang W. Serum Fibrinogen as a Diagnostic and Prognostic Biomarker for Pancreatic Ductal Adenocarcinoma. Pancreatology (2020) 20(7):1465–71. doi: 10.1016/j.pan.2020.06.010 32873483

[66] Simpson RE Yip-Schneider MT Wu H Fan H Liu Z Korc M. Circulating Thrombospondin-2 Enhances Prediction of Malignant Intraductal Papillary Mucinous Neoplasm. Am J Surg (2019) 217:425–8. doi: 10.1016/j.amjsurg.2018.08.026 PMC6380934 30293901

[67] Jiang L Hu LG. Serpin Peptidase Inhibitor Clade A Member 1-Overexpression in Gastric Cancer Promotes Tumor Progression *In Vitro* and Is Associated With Poor Prognosis. Oncol Lett (2020) 20:278. doi: 10.3892/ol.2020.12141 33014156 PMC7520747

[68] Zanini S Renzi S Limongi AR Bellavite P Giovinazzo F Bermano G. A Review of Lifestyle and Environment Risk Factors for Pancreatic Cancer. Eur J Cancer (2021) 145:53–70. doi: 10.1016/j.ejca.2020.11.040 33423007

[69] Fu Y Liu S Zeng S Shen H. The Critical Roles of Activated Stellate Cells-Mediated Paracrine Signaling, Metabolism and Onco-Immunology in Pancreatic Ductal Adenocarcinoma. Mol Cancer (2018) 17(1):62. doi: 10.1186/s12943-018-0815-z 29458370 PMC5817854

[70] Zhang G He P Tan H Budhu A Gaedcke J Ghadimi BM. Integration of Metabolomics and Transcriptomics Revealed a Fatty Acid Network Exerting Growth Inhibitory Effects in Human Pancreatic Cancer. Clin Cancer Res (2013) 19:4983–93. doi: 10.1158/1078-0432.CCR-13-0209 PMC3778077 23918603

[71] Zhao Z Liu W. Pancreatic Cancer: A Review of Risk Factors, Diagnosis, and Treatment. Technol Cancer Res Treat (2020) 19:1533033820962117. doi: 10.1177/1533033820962117 33357065 PMC7768873

[72] Daulton E Wicaksono AN Tiele A Kocher HM Debernardi S Crnogorac-Jurcevic T. Volatile Organic Compounds (VOCs) for the Non-Invasive Detection of Pancreatic Cancer From Urine. Talanta (2021) 221:121604. doi: 10.1016/j.talanta.2020.121604 33076134

[73] McCulloch M Jezierski T Broffman M Hubbard A Turner K Janecki T. Diagnostic Accuracy of Canine Scent Detection in Early- and Late-Stage Lung and Breast Cancers. Integr Cancer Ther (2006) 5(1):30–9. doi: 10.1177/1534735405285096 16484712

[74] Broza YY Khatib S Gharra A Krilaviciute A Amal H Polaka I. Screening for Gastric Cancer Using Exhaled Breath Samples. Br J Surg (2019) 106(9):1122–5. doi: 10.1002/bjs.11294 31259390

[75] Chernov VI Choynzonov EL Kulbakin DE Menkova EN Obkhodskaya EV Obkhodskiy AV. Non-Invasive Diagnosis of Malignancies Based on the Analysis of Markers in Exhaled Air. Diagnostics (Basel) (2020) 10(11):934. doi: 10.3390/diagnostics10110934 PMC7696783 33187053

[76] Gablo NA Prochazka V Kala Z Slaby O Kiss I. Cell-Free microRNAs as Non-Invasive Diagnostic and Prognostic Bio- Markers in Pancreatic Cancer. Curr Genomics (2019) 20(8):569–80. doi: 10.2174/1389202921666191217095017 PMC7290054 32581645

[77] Rashid MH Borin TF Ara R Piranlioglu R Achyut BR Korkaya H. Critical Immunosuppressive Effect of MDSC−Derived Exosomes in the Tumor Microenvironment. Oncol Rep (2021) 45(3):1171–81. doi: 10.1101/2020.03.05.979195 PMC7860000 33469683

[78] Jena BC Mandal M. The Emerging Roles of Exosomes in Anti-Cancer Drug Resistance and Tumor Progression: An Insight Towards Tumor-Microenvironment Interaction. Biochim Biophys Acta Rev Cancer (2021) 1875(1):188488. doi: 10.1016/j.bbcan.2020.188488 33271308

[79] Zhou W Zhou Y Chen X Ning T Chen H Guo Q. Pancreatic Cancer-Targeting Exosomes for Enhancing Immunotherapy and Reprogramming Tumor Microenvironment. Biomaterials (2021) 268:120546. doi: 10.1016/j.biomaterials.2020.120546 33253966

[80] Melo SA Luecke LB Kahlert C Fernandez AF Gammon ST Kaye J. Glypican-1 Identifies Cancer Exosomes and Detects Early Pancreatic Cancer. Nature (2015) 523(7559):177–82. doi: 10.1038/nature14581 PMC4825698 26106858

[81] Zhou CY Dong YP Sun X Sui X Zhu H Zhao YQ. High Levels of Serum Glypican-1 Indicate Poor Prognosis in Pancreatic Ductal Adenocarcinoma. Cancer Med (2018) 7(11):5525–33. doi: 10.1002/cam4.1833 PMC6246926 30358133

[82] Lai X Wang M McElyea SD Sherman S House M Korc M. A microRNA Signature in Circulating Exosomes Is Superior to Exosomal Glypican-1 Levels for Diagnosing Pancreatic Cancer. Cancer Lett (2017) 393:86–93. doi: 10.1016/j.canlet.2017.02.019 28232049 PMC5386003

[83] Servage KA Stefanius K Gray HF Orth K. Proteomic Profiling of Small Extracellular Vesicles Secreted by Human Pancreatic Cancer Cells Implicated in Cellular Transformation. Sci Rep (2020) 10(1):7713. doi: 10.1038/s41598-020-64718-6 32382024 PMC7205864

[84] Castillo J Bernard V San Lucas FA Allenson K Capello M Kim DU. Surfaceome Profiling Enables Isolation of Cancer-Specific Exosomal Cargo in Liquid Biopsies From Pancreatic Cancer Patients. Ann Oncol (2018) 29(1):223–9. doi: 10.1093/annonc/mdx542 PMC6248757 29045505

[85] Si K Xue Y Yu X Zhu X Li Q Gong W. Fully End-to-End Deep-Learning-Based Diagnosis of Pancreatic Tumors. Theranostics (2021) 11(4):1982–90. doi: 10.7150/thno.52508 PMC7778580 33408793

[86] Wang CC Yang TW Sung WW Tsai MC. Current Endoscopic Management of Malignant Biliary Stricture. Medicina (Kaunas) (2020) 56(3):114. doi: 10.3390/medicina56030114 PMC7143433 32151099

[87] Urman JM Herranz JM Uriarte I Rullán M Oyón D González B. Pilot Multi-Omic Analysis of Human Bile From Benign and Malignant Biliary Strictures: A Machine-Learning Approach. Cancers (Basel) (2020) 12(6):1644. doi: 10.3390/cancers12061644 PMC7352944 32575903

[88] Chung WY Correa E Yoshimura K Chang MC Dennison A Takeda S. Using Probe Electrospray Ionization Mass Spectrometry and Machine Learning for Detecting Pancreatic Cancer With High Performance. Am J Transl Res (2020) 12(1):171–9. PMC7013221 32051746

[89] Martinez-Useros J Martin-Galan M Garcia-Foncillas J. The Match Between Molecular Subtypes, Histology and Microenvironment of Pancreatic Cancer and Its Relevance for Chemoresistance. Cancers (Basel) (2021) 13(2):322. doi: 10.3390/cancers13020322 33477288 PMC7829908

[90] Santofimia-Castaño P Iovanna J. Combating Pancreatic Cancer Chemoresistance by Triggering Multiple Cell Death Pathways. Pancreatology (2021) 21(3):522–29. doi: 10.1016/j.pan.2021.01.010 33516629

[91] Mandili G Curcio C Bulfamante S Follia L Ferrero G Mazza E. In Pancreatic Cancer, Chemotherapy Increases Antitumor Responses to Tumor-Associated Antigens and Potentiates DNA Vaccination. J Immunother Cancer (2020) 8(2):e001071. doi: 10.1136/jitc-2020-001071 33115943 PMC7594541

[92] Balluff B Rauser S Ebert MP Siveke JT Höfler H Walch A. Direct Molecular Tissue Analysis by MALDI Imaging Mass Spectrometry in the Field of Gastrointestinal Disease. Gastroenterology (2012) 143(3):544–549.e2. doi: 10.1053/j.gastro.2012.07.022 22820311

[93] Grüner BM Winkelmann I Feuchtinger A Sun N Balluff B Teichmann N. Modeling Therapy Response and Spatial Tissue Distribution of Erlotinib in Pancreatic Cancer. Mol Cancer Ther (2016) 15(5):1145–52. doi: 10.1158/1535-7163.MCT-15-0165 26823494

[94] Prentice BM Hart NJ Phillips N Haliyur R Judd A Armandala R. Imaging Mass Spectrometry Enables Molecular Profiling of Mouse and Human Pancreatic Tissue. Diabetologia (2019) 62(6):1036–47. doi: 10.1007/s00125-019-4855-8 PMC6553460 30955045

[95] Diab M Azmi A Mohammad R Philip PA. Pharmacotherapeutic Strategies for Treating Pancreatic Cancer: Advances and Challenges. Expert Opin Pharmacother (2019) 20(5):535–46. doi: 10.1080/14656566.2018.1561869 30592647

[96] Buettner R Morales C Wu X Sanchez JF Li H Melstrom LG. Leflunomide Synergizes With Gemcitabine in Growth Inhibition of PC Cells and Impairs C-Myc Signaling Through PIM Kinase Targeting. Mol Ther Oncolytics (2019) 14:149–58. doi: 10.1016/j.omto.2019.04.006 PMC6562366 31211245

[97] Phan T Nguyen VH Buettner R Morales C Yang L Wong P. Inhibition of *De Novo* Pyrimidine Synthesis Augments Gemcitabine Induced Growth Inhibition in an Immunocompetent Model of Pancreatic Cancer. Int J Biol Sci (2021) 17:2240–51. doi: 10.7150/ijbs.60473 PMC8241727 34239352

[98] Pham T Shields MA Spaulding C Principe DR Li B Underwood PW. Preclinical Models of Pancreatic Ductal Adenocarcinoma and Their Utility in Immunotherapy Studies. Cancers (Basel) (2021) 13(3):440. doi: 10.3390/cancers13030440 33503832 PMC7865443

[99] Meng M Liu S Wang C Gu X Linghu E Xue X. Mass Spectrum Analysis of Membrane Proteins Reveals That CASK, CD36 and EPB42 Are Differentially Expressed in Pancreatic Adenocarcinoma. Oncol Lett (2020) 20(6):376. doi: 10.3892/ol.2020.12239 33154774 PMC7608047

[100] Olaoba OT Ligali FC Alabi ZO Akinyemi AO Ayinde KS. Of Immune Checkpoint Maladies and Remedies: The Throwing of Jabs in the Oncogenic Ring of PDAC. Biochim Biophys Acta Rev Cancer (2021) 1875(1):188483. doi: 10.1016/j.bbcan.2020.188483 33232723

[101] Krishnamoorthy M Lenehan JG Burton JP Maleki Vareki S. Immunomodulation in Pancreatic Cancer. Cancers (Basel) (2020) 12(11):3340. doi: 10.3390/cancers12113340 PMC7696309 33198059

[102] Edwards P Kang BW Chau I. Targeting the Stroma in the Management of Pancreatic Cancer. Front Oncol (2021) 11:691185. doi: 10.3389/fonc.2021.691185 34336679 PMC8316993

[103] Du W Pasca di Magliano M Zhang Y. Therapeutic Potential of Targeting Stromal Crosstalk-Mediated Immune Suppression in Pancreatic Cancer. Front Oncol (2021) 11:682217. doi: 10.3389/fonc.2021.682217 34290984 PMC8287251

[104] Lee MJ Na K Jeong SK Lim JS Kim SA Lee MJ. Identification of Human Complement Factor B as a Novel Biomarker Candidate for Pancreatic Ductal Adenocarcinoma. J Proteome Res (2014) 13:4878–88. doi: 10.1021/pr5002719 25057901

[105] Kim SH Lee MJ Hwang HK Lee SH Kim H Paik YK. Prognostic Potential of the Preoperative Plasma Complement Factor B in Resected Pancreatic Cancer: A Pilot Study. Cancer biomark (2019) 24:335–42. doi: 10.3233/CBM-181847 PMC13082520 30829612

[106] Shimazaki R Takano S Satoh M Takada M Miyahara Y Sasaki K. Complement Factor B Regulates Cellular Senescence and Is Associated With Poor Prognosis in Pancreatic Cancer. Cell Oncol (Dordr) (2021) 44:937–50. doi: 10.1007/s13402-021-00614-z PMC8338870 34075561

[107] Le Large T Bijlsma MF El Hassouni B Mantini G Lagerweij T Henneman AA. Focal Adhesion Kinase Inhibition Synergizes With Nab-Paclitaxel to Target Pancreatic Ductal Adenocarcinoma. J Exp Clin Cancer Res (2021) 40:91. doi: 10.1186/s13046-021-01892-z 33750427 PMC7941981

[108] Li J Zhang W Gao J Du M Li H Li M. E3 Ubiquitin Ligase UBR5 Promotes the Metastasis of Pancreatic Cancer *via* Destabilizing F-Actin Capping Protein Capza1. Front Oncol (2021) 11:634167. doi: 10.3389/fonc.2021.634167 33777788 PMC7994773

[109] Jiang W Qiao L Han Y Zhang A An H Xiao J. Pancreatic Stellate Cells Regulate Branched-Chain Amino Acid Metabolism in Pancreatic Cancer. Ann Transl Med (2021) 9:417. doi: 10.21037/atm-21-761 33842638 PMC8033345

[110] Ke C Yuan L Xiujiang Y Danjie S. Integrated Analysis of Metabolome in a EUS-FNA Sample With Transcriptome in the TCGA Cohort of Pancreatic Head and Body/Tail Adenocarcinoma. Aging (Albany NY) (2021) 13:8880–94. doi: 10.18632/aging.202700 PMC8034907 33714949

[111] Shi W Zhang C Ning Z Hua Y Li Y Chen L. CMTM8 as an LPA1-Associated Partner Mediates Lysophosphatidic Acid-Induced Pancreatic Cancer Metastasis. Ann Transl Med (2021) 9:42. doi: 10.21037/atm-20-1013 33553335 PMC7859753

[112] Yu S Wu N Zhu J Liu Y Han J. Pyrrolidine Dithiocarbamate Facilitates Arsenic Trioxide Against Pancreatic Cancer *via* Perturbing Ubiquitin-Proteasome Pathway. Cancer Manag Res (2020) 12:13149–59. doi: 10.2147/CMAR.S278674 PMC7764808 33376406

[113] Testoni S Healey AJ Dietrich CF Arcidiacono PG. Systematic Review of Endoscopy Ultrasound-Guided Thermal Ablation Treatment for Pancreatic Cancer. Endosc Ultrasound (2020) 9(2):83–100. doi: 10.4103/eus.eus_74_19 32295966 PMC7279078

[114] Gao S Pu N Yin H Li J Chen Q Yang M. Radiofrequency Ablation in Combination With an mTOR Inhibitor Restrains Pancreatic Cancer Growth Induced by Intrinsic HSP70. Ther Adv Med Oncol (2020) 12:1758835920953728. doi: 10.1177/1758835920953728 32973929 PMC7491221

[115] Jiang W Li X Dong S Zhou W. Betulinic Acid in the Treatment of Tumour Diseases: Application and Research Progress. BioMed Pharmacother (2021) 142:111990. doi: 10.1016/j.biopha.2021.111990 34388528

[116] Chiu CF Chang HY Huang CY Mau CZ Kuo TT Lee HC. Betulinic Acid Affects the Energy-Related Proteomic Profiling in Pancreatic Ductal Adenocarcinoma Cells. Molecules (2021) 26(9):2482. doi: 10.3390/molecules26092482 33923185 PMC8123215

